# Construction of a Microfluidic Platform With Core-Shell CdSSe@ZnS Quantum Dot-Encoded Superparamagnetic Iron Oxide Microspheres for Screening and Locating Matrix Metalloproteinase-2 Inhibitors From Fruits of *Rosa roxburghii*

**DOI:** 10.3389/fnut.2022.869528

**Published:** 2022-04-14

**Authors:** Yi Tao, Meiling Pan, Fei Zhu, Qing Liu, Ping Wang

**Affiliations:** College of Pharmaceutical Science, Zhejiang University of Technology, Hangzhou, China

**Keywords:** microfluidic, magnetic microspheres, *Rosa roxburghii*, matrix metalloproteinase-2, ligand fishing

## Abstract

The microfluidic platform is a versatile tool for screening and locating bioactive molecules from functional foods. Here, a layer-by-layer assembly approach was used to fabricate core-shell CdSSe@ZnS quantum dot encoded superparamagnetic iron oxide microspheres, which served as a carrier for matrix metalloproteinase-2. The matrix metalloproteinase-2 camouflaged magnetic microspheres was further incorporated into a homemade microfluidic platform and incubated with extracts of fruits of *Rosa roxburghii*. The flow rate of the microfluidic platform was tuned. The major influencing parameters on ligand binding, such as dissociate solvents, incubation pH, ion strength, temperature, and incubation time were also optimized by using ellagic acid as a model compound. The specific binding ligands were sent for structure elucidation by mass spectrometry. The absolute recovery of ellagic acid ranged from 101.14 to 102.40% in the extract of *R. roxburghii* under the optimal extraction conditions. The linearity was pretty well in the range of 0.009–1.00 mg·ml^−1^ (*R*^2^ = 0.9995). The limit of detection was 0.003 mg·ml^−1^. The relative SDs of within-day and between-day precision were <1.91%. A total of thirteen ligands were screened out from fruits of *R. roxburghii*, which were validated for their inhibitory effect by enzyme assay. Of note, eleven new matrix metalloproteinase-2 inhibitors were identified, which may account for the antitumor effect of fruits of *R. roxburghii*.

## Introduction

*Rosa roxburghii* Tratt (RRT), which mainly grows in Guizhou province (1), has attracted intensive attention owing to its diverse biological properties such as antitumor (2, 3), antiatherogenic (4), antioxidant (5), antibrowning (6), hyperglycemic (7), radioprotection (8, 9), and so on. Recently, the fruits of RRT have been transformed into functional foods such as standardized juice and beverage products. Triterpenoids, phenolics, and polysaccharides were reported to be the major components of RRT fruits (10). Although many endeavors have shown that extracts of RRT fruits own excellent antitumor effects (11), the components which account for the antitumor property of RRT fruits remain unclear.

Ligand fishing is a feasible strategy for discovering bioactive components from the complex mixture of natural products (12). A series of nanomaterials have been used as support for the drug targets. For instance, Hsp 90α functionalized InP/ZnS quantum dots (QDs) embedded mesoporous nanoparticles were employed for ligand fishing from *Curcuma longa* L (13). Moreover, PTP1B displayed *Escherichia coli* cells can be used for ligand fishing from the extracts of *Rhodiola rosea* (14). Besides, magnetic microspheres (MSs) have recently been examined as a very useful material for ligand fishing (15, 16). The surface of MSs could be easily modified and coated with enzymes or proteins that enable them to bind to other biologically active compounds.

The rapid development of microfluidics technology (17) has promoted new innovations in the field of ligand fishing. For instance, a three-phase-laminar-flow-chip was developed for fishing antitumor ingredients by G-quadruplex recognition from *Macleaya cordata* seeds extracts (18, 19). Precise manipulation of fluids and MSs in a microfluidic chip will not only accelerate the speed of ligand fishing but also reduce the expenditure of the expensive target protein and other solvents. Importantly, microfluidic devices are one of the most promising platforms to mimic *in vivo* like conditions (20).

**Figure 1:**
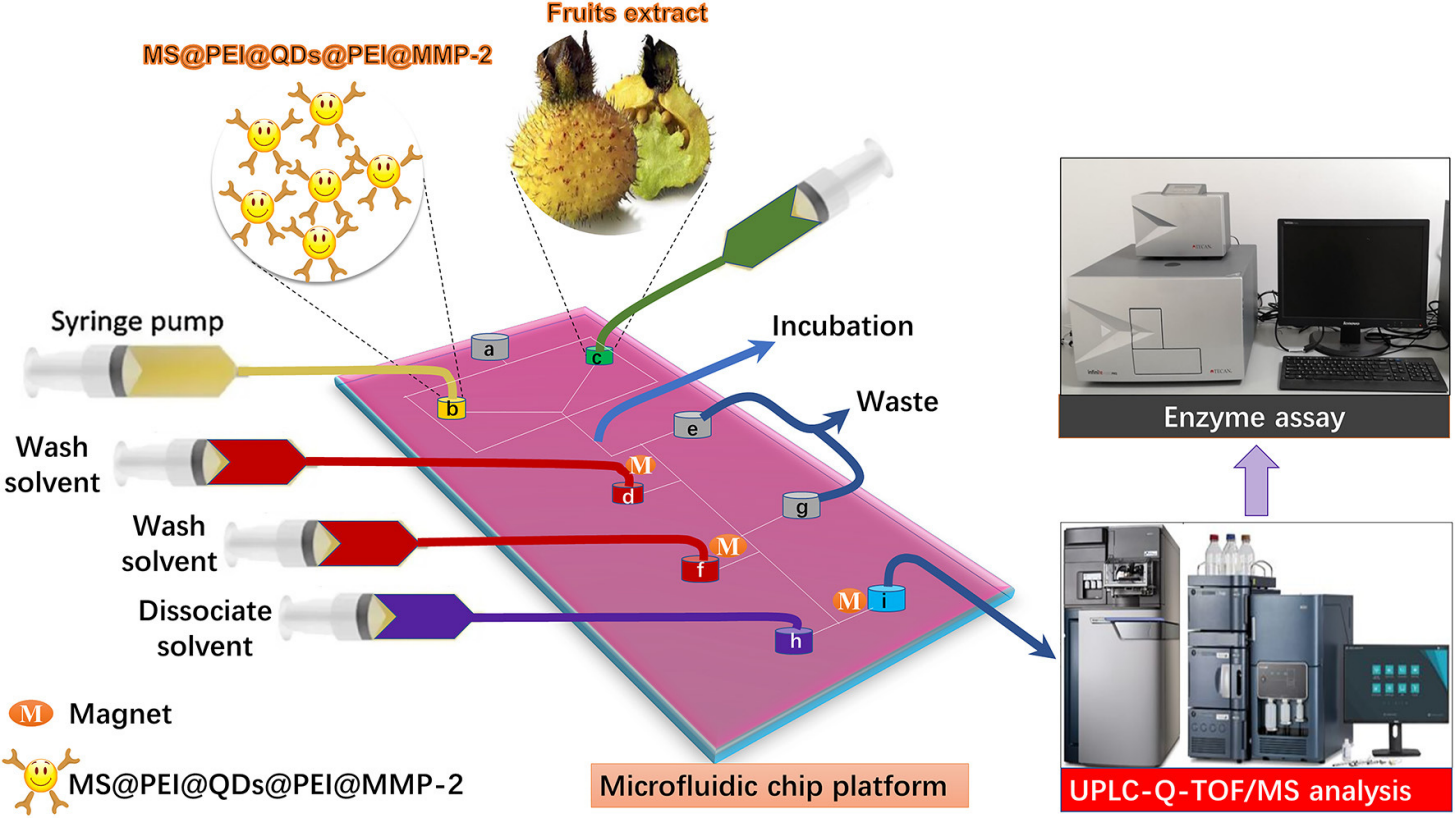
Schematic of microfluidic chips-based matrix metalloproteinases inhibitors recognition system. MS@PEI@QDs@PEI@MMP-2 and extract of *Rosa roxburghii* were infused into the channel of the microfluidic chip and incubated for a period. Then, unspecific binding compounds were washed to the waste by using magnetic separation. The dissociated solvent was pumped into the channel to dissociate the MMP-2 binding ligands. The ligands were sent for UPLC-Q-TOF/MS analysis and validated by using an enzyme assay.

Quantum dots (QDs) are ideal candidate fluorophores for optical visualization because of their outstanding fluorescent properties. Herein, to visualize the flow of the MSs in the microfluidic channels, hydrophobic cadmium selenium sulfide/zinc sulfide (CdSSe/ZnS) QDs were first loaded onto the surface of magnetic polystyrene spheres via the modified layer-by-layer assembly. After that, the target protein was immobilized onto the polyethylenimine (PEI) modified surface of MS@QDs. As a showcase, matrix metallopeptidase 2 (MMP-2), which is highly expressed in the most of tumors, namely, breast, prostate, and bowel cancers (21), is selected as the target protein. The schematic of incorporation of the MS@PEI@QDs@PEI@MMP-2 into a homemade microfluidic platform for ligand fishing from RRT fruits is present in Figure 1. Briefly, MS@PEI@QDs@PEI@MMP-2 and extract of *R. roxburghii* were infused into the channel of the microfluidic chip and incubated for a period. Then, unspecific binding compounds were washed to the waste by using magnetic separation. The dissociated solvent was pumped into the channel to dissociate the MMP-2 binding ligands. The ligands were sent for UPLC-Q-TOF/MS analysis and validated by using an enzyme assay.

## Materials and Methods

### Materials

The raw materials of *R. roxburghii* were collected from Guiyang County of Guizhou Province and authenticated by Prof. Ping Wang. Voucher specimens were stored in the Zhejiang University of Technology (No. RR2021041). Recombinant human matrix metalloproteinase-2 (expressed in HEK 293 cells), 1-(3-dimethyl-aminopropyl)-3-ethyl-carbodiimide (EDC), *N*-hydroxysuccinimide (NHS), and PEI (molecular weight: 750,000 Da) were purchased from Sigma Co. 2-(N-morpholino) ethanesulfonic acid (MES) was obtained from Shanghai Yien Chemical Technology Co. Oil-soluble core-shell CdSSe/ZnS QDs (5 mg·ml^−1^) with an emission wavelength at 594 nm and carboxyl terminated MSs (diameter: 5.53 μm and volume: 10 ml) were obtained from Hebei Langfei Biotechnology Co.

Standard substances, namely, ellagic acid, loganic acid, corilagin, oreganol A, kajiichigoside F_1_, zebirioside O, niga-ichigoside F_1_, and quadranoside VIII, were purchased from Weikeqi Bio-technology Co. Peduncloside and valerenic acid were obtained from Shanghai Yuanye Bio-Technology Co. Potentillanoside A was supplied by Shanghai Tauto Biotech Co. Medicagenic acid was obtained from Chengdu Biopurify Phytochemicals Co. Rosamultin and batimastat were purchased from MedChemExpress Co. Purities of all the reference compounds were >98%. ELISA kit for testing human matrix metalloproteinase-2 was obtained from Shanghai Easy Biotech Co. UPLC-grade acetonitrile was purchased from Merck Co. A 96-well microtiter plate was obtained from Corning Co.

### Apparatus

The analytical apparatus included a JEM-1200EX transmission electron microscopy (NEC Co), a Scanning electron microscope (SEM, Hitachi S4800), a Confocal laser scanning microscopy (Zeiss Co.), an XPert PRO X-ray Diffractometer (PANalytical Co.), a PPMS-9 vibration sample magnetometer (Quantum Design Co.), a Jasco-4100 Fourier transform infrared spectroscopy (Jasco Co.), a Waters SYNAPT G2 quadrupole-ion mobility-time-of-flight mass spectrometry (Waters MS Technologies Co.), a PB-10 Sartorius pH meter (Sartorius Co.), a Tecan's Infinite 200 PRO system (Tecan Co.), and a Milli-Q Water Purification System (Millipore Co.).

### Fabrication of Microfluidic Chips

The microfluidic chip was prepared by employing a standardized soft lithography technology (22, 23). Briefly, a new silicon wafer was rinsed with ethanol and dried with a stream of air. To prevent surface defects, forceps were used to handle the wafer. The wafer was transferred to a spin coater for 10 s at 600 rpm, followed by 30 s at 3,000 rpm, and then covered with a SU-2025 negative photoresist. Due to the photosensitivity of the photoresist, the room lights were turned off. After that, the photoresist-coated silicon wafer was transferred to a hot plate for “soft-bake” at 95°C for three min. Then, the photoresist coated silicon wafer was removed from the hot plate and allowed to cool for 1 min. A prefabricated mask with the desired pattern was placed on top of the photoresist-covered silicon wafer, which was then exposed to ultraviolet irradiation (350–450 nm) for 20 s. Subsequently, the silicon wafer was transferred to the hot plate for “hard-bake” at 110°C for six min. The room lights were turned on. The silicon wafer was carefully rinsed with a SU-8 photoresist developer three times and then rinsed with ethanol and air-dried. After that, polydimethylsiloxane (PDMS) elastomer solution and curing agent were mixed in a 10:1 weight ratio and then centrifuged at 900 rpm for 5 min to remove air bubbles. PDMS was poured onto the patterned silicon wafer in Petri dish and cured overnight at room temperature to form a complementary elastomeric stamp. Finally, PDMS was carefully removed from the silicon wafer. A razor edge was used to cut the stamp to the desired size. The homemade microfluidic chip is shown in Supplementary Figure 1. The width and height of the channels in the chip were 400 and 100 μm, respectively.

### Preparation of MS@PEI@QDs@PEI@MMP-2

First, 600 μl aliquots of MSs were pipetted into a centrifuge tube, and the supernatant was removed by magnetic separation. The MS was further washed with MES buffer solution (10 mM, pH = 5) three times, and the supernatant was abandoned. A freshly prepared carbodiimide (EDC) solution (3 g·l^−1^) and PEI solution (80 g·l^−1^) were added to the MS successively, vortexed, and ultrasonically dispersed. After rotating for 3 h, the supernatant of the mixture solution was removed by magnetic separation. The remaining PEI-modified MS was washed 3 times with ultrapure water.

Second, 3 mg of MS@PEI was transferred to a centrifuge tube and washed with ethanol three times. Then, 1 ml aliquot of the CdSSe/ZnS QDs solution in chloroform/n-butanol (volume ratio, 1:20) was added into the centrifuge tube, vortexed, and rotated in the dark for 30 min. After that, the supernatant was removed by magnetic separation. Finally, MS@PEI@QDs were obtained and washed with ethanol 3 times and dispersed in ultrapure water.

Third, 1 ml aliquot of PEI solution (9 g·l^−1^) was prepared and added to the MS@PEI@QDs. The mixture solution was vortexed and then rotated in the dark for 30 min. The supernatant was removed by magnetic separation. Subsequently, MS@PEI@QDs@PEI was washed with ultrapure water three times. A total of 4 ml EDC (1 mg·ml^−1^) and 10 ml NHS (1 mg·ml^−1^) solution were added to 1 ml matrix metalloproteinase-2 solution, and reacted for 30 min to activate the carboxylic acid groups of matrix metalloproteinase-2. After that, this solution was rapidly added into 1.0 ml MS@PEI@QDs@PEI solution and agitated for 12 h to obtain MS@PEI@ QDs@PEI@MMP-2.

### Characterization of MS@PEI@QDs@PEI@MMP-2

The surface of blank MS and modified MS were characterized by using transmission electron microscopy (TEM) and Fourier transform infrared spectroscopy. Magnetization was recorded at room temperature in a vibration sample magnetometer. The crystalline structure of MSs was identified by using a powder X-ray diffractometer. Confocal laser scanning microscopy was used to visualize the distribution of MS@PEI@QDs@PEI@MMP-2.

Experiments with varying amounts of MS@PEI@QDs@PEI (50, 100, 150, 200, and 250 μl) and a constant amount of matrix metalloproteinase-2 (200 μg) were carried out to investigate the amount of protein efficiently immobilized on the MS. Samples of the supernatants were withdrawn and analyzed for remaining protein by using the Bradford method and the calibration curve was prepared using solutions of bovine serum albumin. The protein immobilized on the MS was determined.

### Optimization Conditions for Microfluidic Chip System-Based Ligand Fishing

All ligand fishing experiments were performed on the microfluidic chip system. The microfluidic chip system consisted of six modules, namely, a microscope stand, a light-emitting diode surface light source, a charge-coupled device (CCD) camera, a temperature controller, an automated x-y-z translation stage (Wuhan Mesovision Biotechnology Co.) for controlling the movement of the microfluidic chip, and four syringe pumps (Baoding Lange Constant Current Pump Co.). Before use, the microfluidic chip was first treated with 1% tween 80 in n-hexadecane (v/v) through port **a** to make the whole chip surface hydrophobic. A well-known matrix metalloproteinase-2 inhibitor, ellagic acid (24), was employed to select the best experimental conditions. Under the monitoring of the top-view CCD camera, MS@PEI@QDs@PEI@MMP-2 were infused into the upper left port **b**, whereas extract of *R. roxburghii* was infused into upper right port **c** at an appropriate flow rate to allow the solution to meet the MS@PEI@QDs@PEI @MMP-2 at the junction. The mixture was incubated in the channel for a period. Then, 500 μl aliquots of wash solvent (PBS buffer) were infused into the port **d**. At the turning point, a small ring magnet trapped the MS@PEI@QDs@PEI@MMP-2. Unspecific binding ligands were eluted to the waste port **e**. Again, 500 μl aliquots of wash solvent (PBS buffer) were infused into the port **f** and unspecific binding ligands were eluted to the waste port **g**. Finally, dissociate solvent was infused into the port **h**, the binding ligands were collected at port **i** and then sent for UPLC-Q-TOF/MS analysis.

Several parameters were investigated to optimize the screening conditions. At first, flow rates of the pump (10, 30, 50, 70, 90, and 120 μl/min) were investigated for optimization of the washing procedure. Second, different dissociation solvent was interrogated including methanol-water (10, 30, 50, 70, and 90%, v/v) and acetonitrile-water (10, 30, 50, 70, and 90%, v/v). Third, a gradient pH (pH 5.6, 6.2, 6.8, 7.4, and 8.0) and a gradient concentration (10, 50, 100, 250, and 500 mM, pH 7.4) of phosphate buffers were applied to study the effect of pH and ion strength of the incubation solution. Finally, incubation temperature (25, 30, 37, 45, and 50°C) and incubation time (10, 20, 30, 40, and 50 min) were also investigated for optimization of the screening procedure.

### Validation of Microfluidic Chip System-Based Ligand Fishing Method

The ligand fishing was performed as described above. The specificity of the method was corroborated by detecting the interference of negative compound (loganic acid, 0.1 mg·ml^−1^) at the retention times of the positive compound (ellagic acid, 0.1 mg·ml^−1^). To obtain denatured MS@PEI@QDs@PEI@MMP-2, the prepared MS@PEI@QDs@PEI@MMP-2 was boiled in 100°C water for 10 min. The linearity was analyzed by plotting the peak area of the ellagic acid against a series of concentrations (0.009–1.00 mg·ml^−1^). The lower limit of quantification was determined as the analytical concentration at an S/N ratio of 10. The intra- and interday precisions of the method were performed by repeated analyzing standard samples (*n* = 6) on the same day or on three consecutive days. The repeatability was interrogated by analyzing the same sample six times. Stability study was carried out with sample solution at 0, 2, 4, 8, 16, and 24 h. The recovery of the method was carried out by analyzing the peak area of ellagic acid (low, medium, and high concentration) spiked extract of *R. roxburghii* and the peak area of ellagic acid in the routine extract of *R. roxburghii*. The analysis was performed on an ACQUITY UPLC HSS T3 analytical column (1.8 μm, 100 × 2.1 mm i.d.). 0.1% Formic acid-water (A) and methanol (B) were selected as the mobile phase at a flow rate of 0.3 ml/min. The UV spectra were monitored at 254 nm. The injection volume was set to 2 μl.

### Ligand Fishing From Extracts of *Rosa roxburghii*

The dried fruits of *R. roxburghii* were first ground into powder and sieved (60 mesh). After that, 5.0 g of powder was weighed and ultrasonicated in 50 ml of 70% ethanol-water for 30 min. The extraction was carried out two times. The extracts were combined and evaporated under a vacuum. Ligand fishing was performed according to the procedure as described above. The dissociated ligands were analyzed by using UPLC-Q-TOF/MS. The chromatographic separation was carried out on an ACQUITY UPLC HSS T3 analytical column (1.8 μm, 100 × 2.1 mm i.d.) with the column temperature set at 30°C. The mobile phase consisted of water containing 0.1% (v:v) formic acid (A) and methanol (B). A gradient program was used according to the following profile: 0–15 min, 5–55%B; 15–30 min, 55–95%B; 30–32 min, 95–100%B; and 32–35 min, 100–5%. The flow rate was 0.3 ml/min, and the injection volume was 2 μl. The UV spectra were recorded from 190 to 400 nm while the chromatogram was acquired at 254 nm. The acquisition parameters for mass spectrometry analyzes were as below: collision gas, ultrahigh-purity helium (He); nebulizing gas, high purity nitrogen (N_2_); ion source temperature 120°C; cone voltage −30 V; desolvent gas flow rate 800 l/h; desolvation temperature 350°C; and mass range recorded *m/z* 50–1,500. Mass data analysis was performed by using MassLynx software (version 4.1, Waters MS Technologies Co).

### Matrix Metalloproteinase-2 Inhibitory Assay

The inhibitory effects of binding ligands were evaluated as below. Batimastat served as a positive control. Test components were dissolved in the buffer to yield a gradient of concentrations between 1 and 200 μM. A total of 50 μl of test components and 100 μl of the enzyme solution were added to a 96-well plate and incubated for 30 min at 37°C. Afterward, the solution in the 96-well plate was discarded. The plate was dried on water adsorbing paper. A total of 200 μl of wash solution was added to each well and incubated for 30 s. Again, the solution in the 96-well plate was abandoned. The plate was dried on water adsorbing paper. This step was carried out five times. A total of 50 μl aliquots of solution A and 50 μl aliquots of solution B were added to each well and kept in a dark place for 15 min. As a result, 50 μl aliquots of stopping solution were pipetted into each well. The plate was monitored at the absorbance of 450 nm by using an automatic microplate reader.

### Molecular Docking

The chemical structures of the ligands were imported into ChemBio3D Ultra 14.0 for energy minimization and saved in mol2 format. The minimum root-mean-square gradient was set to 0.001. The optimized small molecules are imported into AutodockTools-1.5.6 for hydrogenation, calculating the charge, assigning the charge, setting the rotatable bond, and saving it as “pdbqt” format. The protein structure of MMP-2 (PDB ID: 1CK7) was downloaded from the PDB database. Pymol 2.3.0 software was used to remove crystal water and original ligands. AutoDock Vina 1.1.2 was employed for docking, MMP-2-related parameters are set as below: center_x = 59.511, center_y = 96.28, and center_z = 147.189; search space: size_x: 126, size_y: 126, and size_z: 126 (the spacing between each grid point is 0.375Å), exhaustiveness: 10, and other parameters are default settings.

## Results and Discussion

### Morphological Characterization

**Figure 2:**
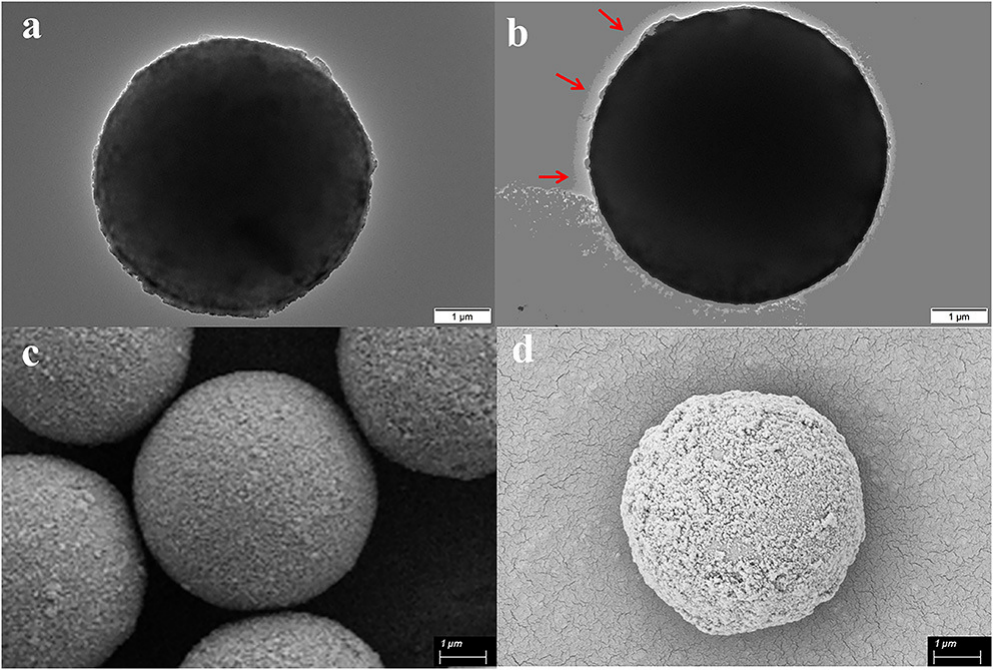
Characterization of magnetic microspheres and MS@PEI@QDs@PEI @MMP-2. **(a)** TEM image of blank magnetic microspheres. **(b)** TEM image of MS@PEI@QDs@PEI@MMP-2. **(c)** The SEM picture of blank magnetic microspheres. **(d)** The SEM picture of MS@PEI@QDs@PEI@MMP-2.

As is shown in Figure 1, the successful immobilization of matrix metalloproteinase-2 onto the MS@PEI@QDs@PEI was clearly revealed from the TEM images of the MSs before (Figure 2A) and after conjugation (Figure 2B). Scanning electron microscope images also showed that the surface of MSs was covered with a white layer (Figures 2C,D). FT-IR analysis provides direct proof for the immobilization of MMP-2 onto the MS@PEI@QDs@PEI. As is displayed in Supplementary Figure 2, the appearance of the C–N stretching band at (1,089.52 cm^−1^) showed that the conjugation took place in a covalent manner through an amide linkage.

As is displayed in Supplementary Figure 3, the surface of the original MS shows a weak negative charge (−2.18 mV). After being modified with PEI, the surface potential becomes positive (+73.38 mV), indicating PEI was successfully attached to the MS surface. After loading the CdSSe/ZnS QDs, the positive potential of the MS@PEI@QDs surface was reduced (+68.67 mV), which is ascribed to that some of the amine groups are coordinated with CdSSe/ZnS QDs. When the PEI molecules were further adsorbed onto the surface of the MS@PEI@QDs, the zeta potential of MS@PEI@QDs@PEI surface was restored to a higher level (71.03 mV), revealing the surface of the MS@PEI@QDs@PEI is rich in amine functional groups. The loading of MMP-2 onto the surface lead to the drop in the zeta potential (53.99 mV), due to the consumption of the amine group.

**Figure 3:**
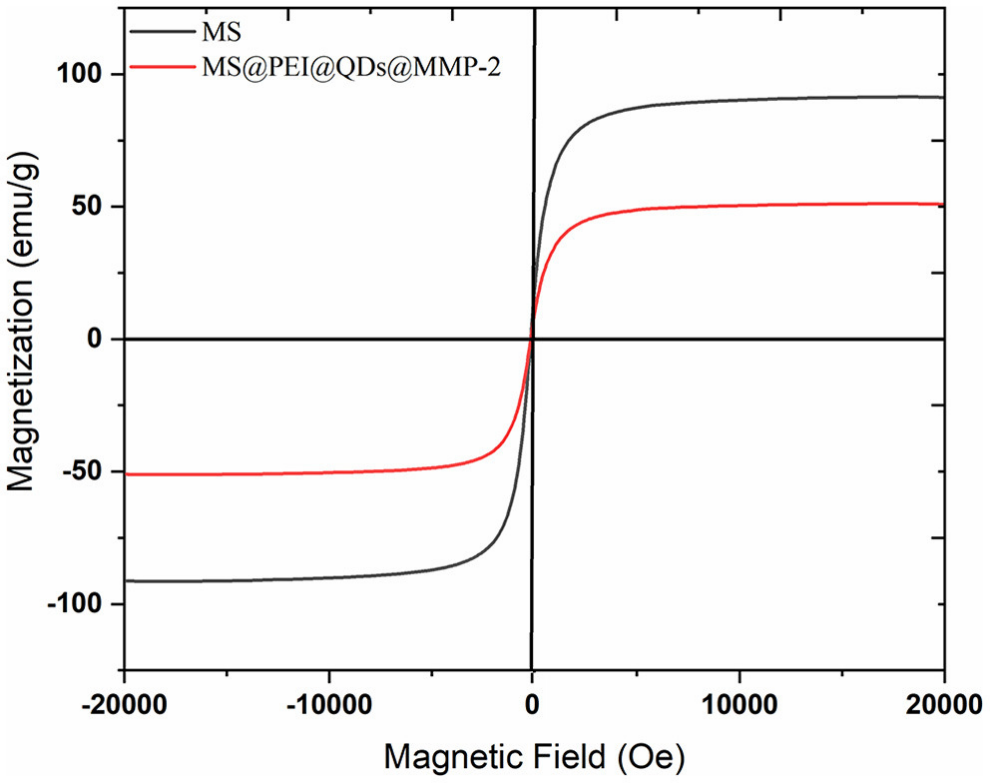
Magnetization curves of blank magnetic microspheres (black line) and MS@PEI@QDs @PEI@MMP-2 (red line).

The main peaks of the X-ray diffraction (XRD) of the MSs (black line) match well with the standard magnetite Fe_3_O_4_ XRD spectrum are displayed in Supplementary Figure 4. The peaks at 25.04°, 38.6°, and 48.76° correspond to the XRD spectrum of CdSSe/ZnS. Magnetization curves of blank MS and MS@PEI@QDs@PEI@MMP-2 are shown in Figure 3. The maximum saturation magnetization (51.15 emu/g) of MS@PEI@QDs@PEI@MMP-2 is a little less than that (91.44 emu/g) of blank MSs. The amounts of MSs were varied to determine the optimal ratio for immobilization of matrix metalloproteinase-2. As is shown in Supplementary Figure 5, when 150 μl MSs per 100 μg matrix metalloproteinase-2 were arranged, the largest percentage of conjugated protein was achieved. Thus, a ratio of 150 μl MSs per 100 μg matrix metalloproteinase-2 was chosen for subsequent studies. Supplementary Figure 6 shows the confocal images of the obtained MS@PEI@QDs@PEI@MMP-2. It can be seen that there is a uniform red circle on the surface. The color halo further validated that the CdSSe/ZnS QDs were successfully loaded on the MS surface and were uniformly distributed on the MS surface. Meanwhile, the MS@PEI@QDs@PEI@MMP-2 were uniformly dispersed without obvious agglomeration, indicating that they have good dispersibility in an aqueous solution.

### Optimization of Screening Condition

**Figure 4:**
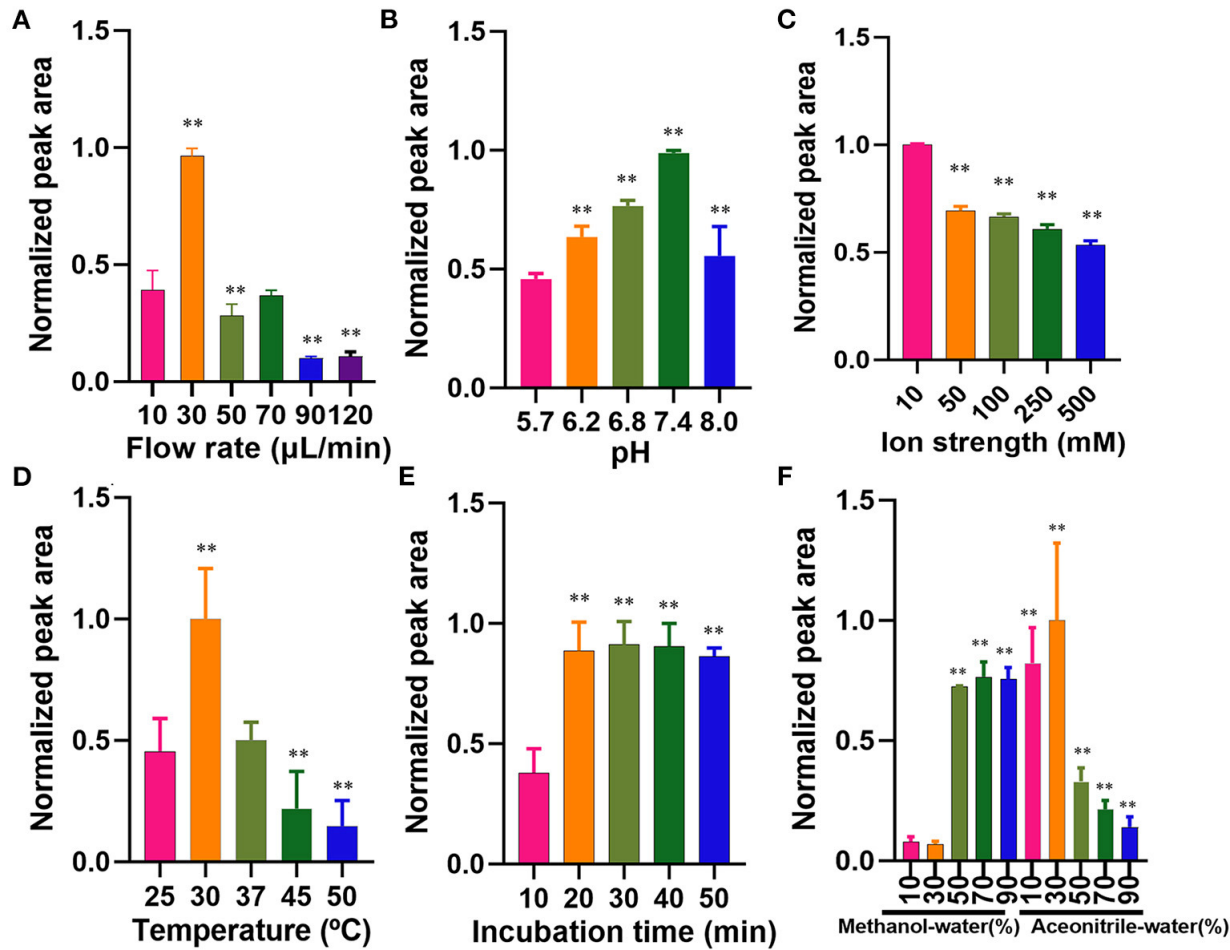
Optimization of parameters affecting the microfluidic chip-based ligand screening system. **(A)** Flow rate, **(B)** pH profiles, **(C)** ionic strength profiles, **(D)** temperature profiles, **(E)** incubation time profiles, and **(F)** dissociated solvent. The symbol ** indicates *P* < 0.05 as compared with the first column.

The flow rate of the microfluidic chip-based ligand fishing system is of great importance for the binding between the ligand and matrix metalloproteinase-2. As is presented in Figure 4A, a flow rate of 30 μl/min was the most suitable rate for ligand fishing. Non-covalent interaction between ligand and matrix metalloproteinase-2 is mainly driven by electrostatic interactions, the ionic strength and pH are of great importance to the interaction. As is shown in Figure 4B, the increase of pH from 5.7 to 7.4 led to an obvious decrease in the peak area of ellagic acid. Further increasing the pH value to 8.0 resulted in the drop of the peak area of ellagic acid. The optimal pH for the incubation is 7.4. The effect of ionic strength was investigated over a broad range of PBS concentrations (10–500 mM). As is displayed in Figure 4C, excessive PBS led to a significant decrease in binding between ellagic acid and MS@PEI@QDs@PEI @MMP-2. Thus, a PBS concentration of 10 mM was selected for the next experiment.

The incubation temperature was also investigated by varying temperatures ranging from 25 to 50°C. As is shown in Figure 4D, 30°C may be the suitable temperature for the incubation. Kato et al. (25) found that the reaction temperature was 37°C for human matrix metalloproteinase-2. In our experiment, the shift of 30–37°C suggested a significantly decreased binding efficiency. As is displayed in Figure 4E, an incubation time of 20 min was sufficient for the interaction between matrix metalloproteinase-2 and ellagic acid. Increasing the incubation time from 20 to 50 min exerted no significant effect on binding efficiency. Therefore, the incubation temperature and time were set to 30°C and 20 min.

The washing step plays a crucial role in ligand fishing. Different proportions of acetonitrile-water and methanol-water (10, 30, 50, 70, and 90% v/v), were arranged as denature solvents and investigated. The data of wash solvent is present in Figure 4F. The denaturation effect of 30% (v/v) acetonitrile-water was the best and was thus assigned as the denaturing solvent.

### Method Validation

To investigate the specificity of the method, both positive control ellagic acid and negative control loganic acid were selected. As is presented in Supplementary Figures 7A,B, only the peak of ellagic acid was observed after ligand fishing. No interference of the negative control loganic acid was observed. The method showed good specificity.

**Figure 5:**
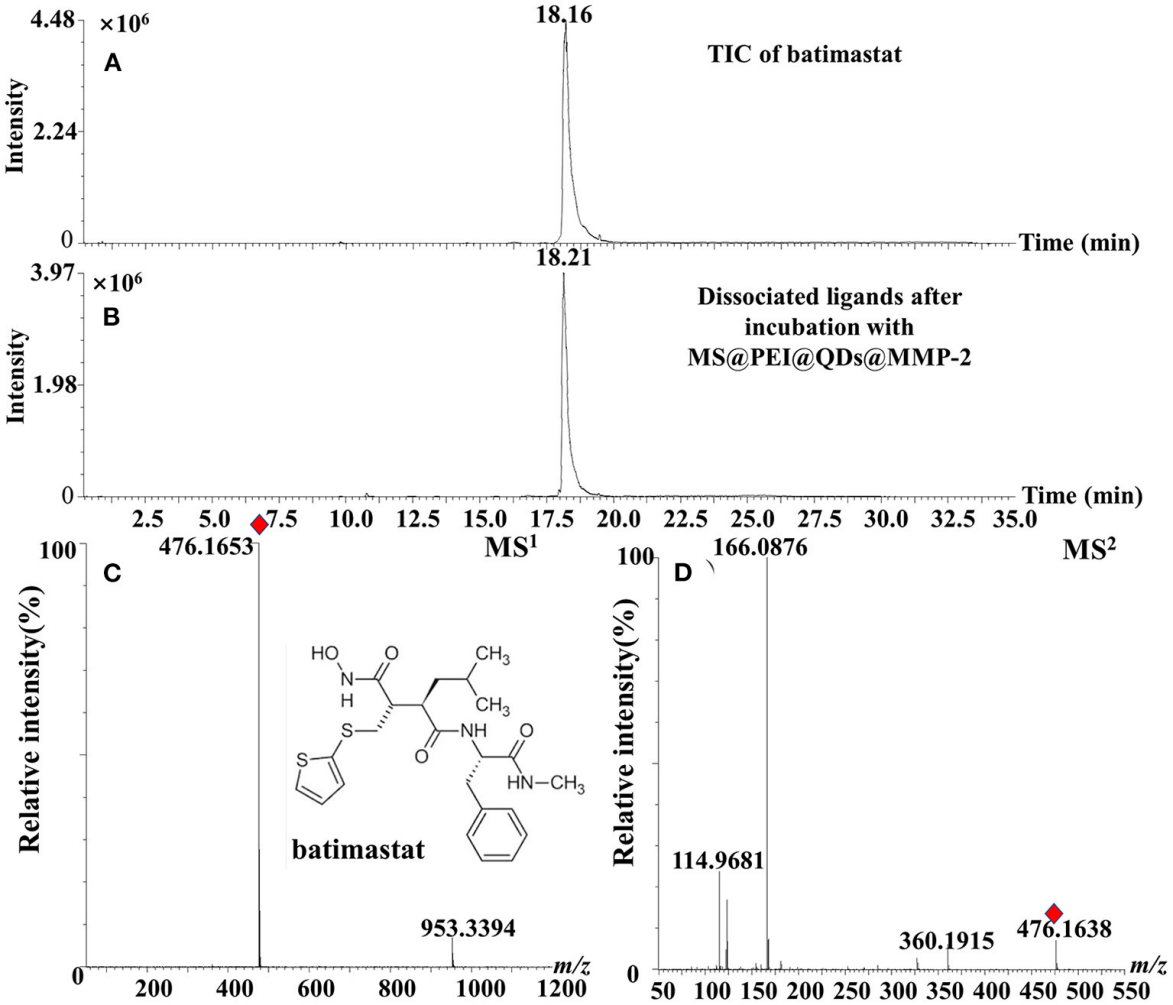
TIC chromatograms of **(A)** batimastat, **(B)** dissociated solution after incubation with MS@PEI@QDs@PEI@MMP-2, **(C)** MS^1^ chromatogram, and **(D)** MS^2^ chromatogram of batimastat.

The calibration curve of ellagic acid showed good linearity in the range of 0.009–1.00 mg·ml^−1^ (*R*^2^ = 0.9995). The limit of detection and limit of quantification of ellagic acid was determined to be 0.003 mg·ml^−1^ and from 0.009 mg·ml^−1^. The relative SD (RSD) values of intraday and interday precisions were <1.91%. The RSD values of stability of ellagic acid were determined to be 2.53%. As is displayed in Supplementary Table 1, the average recoveries of ellagic acid were from 101.14 to 102.40% with RSD values from 1.21 to 2.13% for *R. roxburghii*. The RSD% values of repeatability of the method were no more than 2.98%. Moreover, the positive control batimastat was employed to interrogate the method. As is present in Figures 5A,B, the peak of batimastat was observed in the dissociated solution after microfluidic chip-based ligand fishing. The MS^1^ and MS^2^ information of batimastat (see Figures 5C,D) was in agreement with that of the literature (26). The extraction yield of batimastat was calculated as 76.6%. Overall, the developed microfluidic chip-based ligand fishing method was sensitive and robust.

### Ligands Fishing From Extracts of *Rosa roxburghii*

**Figure 6:**
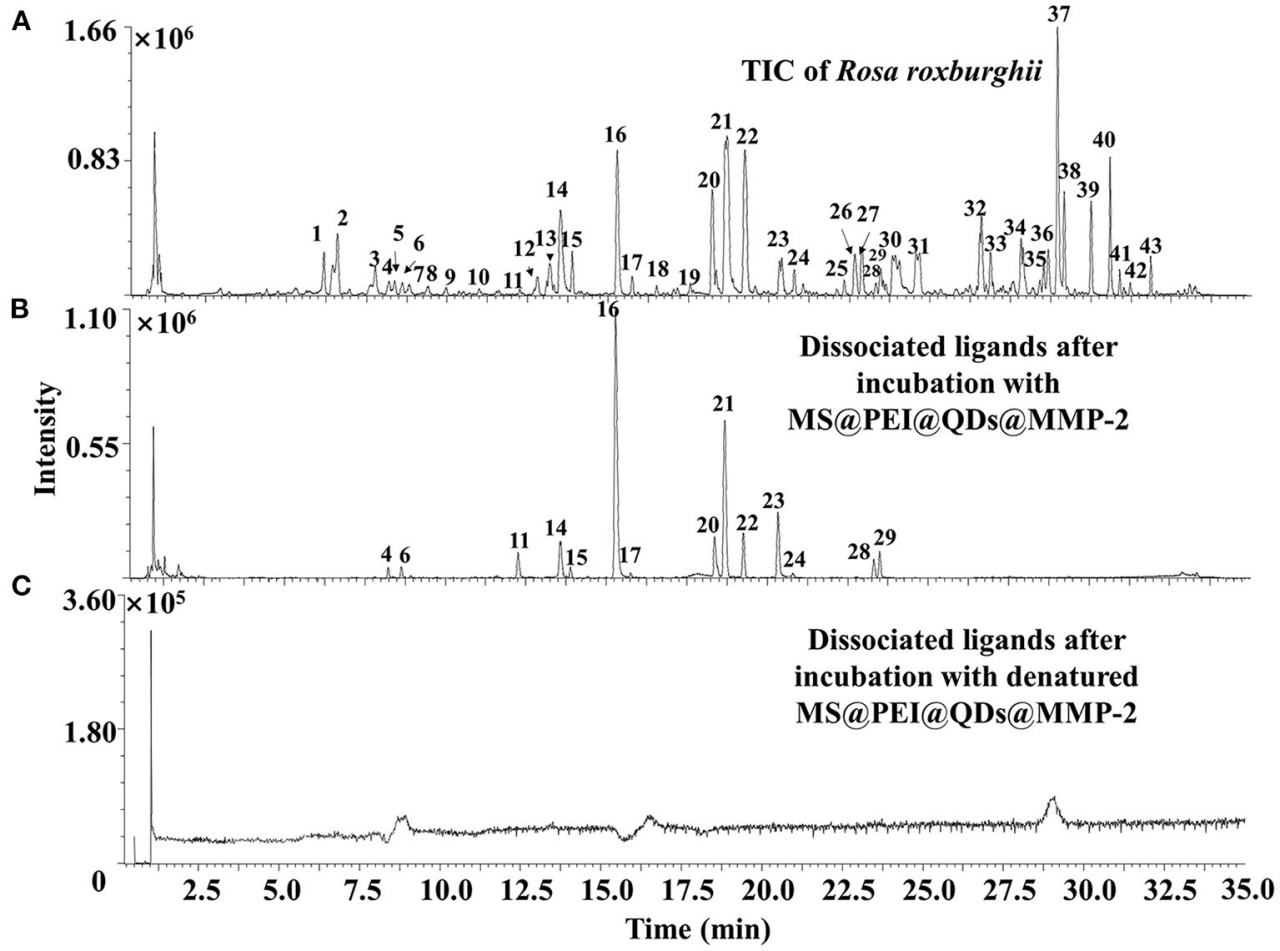
TIC of **(A)** crude extract of *Rosa roxburghii* and **(B)** dissociated MMP-2 binding ligands after incubation with MS@PEI@QDs@PEI@MMP-2 or **(C)** denatured MS@PEI@QDs@PEI@MMP-2.

Figure 6A shows the total ion chromatograms of the crude extract of *R. roxburghii*, while Figure 6B presents the dissociated ligands after incubation with MS@PEI@ QDs@PEI@MMP-2. To exclude the non-specific binding, the TIC of dissociated solution after incubation with denatured MS@PEI@QDs@PEI@MMP-2 is presented in Figure 6C. It can be seen that none of the compounds were bound to the denatured MS@PEI@QDs@PEI@MMP-2. Detailed chemical information of the fourteen ligands is listed in Table 1. The structures of thirteen ligands (see Supplementary Figure 8) were elucidated by analyzing and comparing their retention times, UV data, and MS data with those of standard compounds. The compound corresponding to peaks 4 showed a parent ion at *m/z* 437.1101 [M-H]^−^ and a daughter ion at *m/z* 300.9981 in negative MS^2^ mode. In comparison with standard substance and the literature (27), peaks 4 was deduced as oreganol A. Peak 6 at the retention time of 8.63 min showed an [M-H]^−^ ion at *m/z* 633 and major fragment ions at 301 [M-H-332]^−^, were consistent with the loss of gallic acid and one glucose group, bonded to hexahydroxydiphenoyl group unit. Compared with the literature (28) and standard compound, peak 6 was unambiguously identified as corilagin. The classification of compounds into ellagic acid or quercetin-based conjugates was made based on typical for these structures fragments ions appearing in MS/MS spectra. Fragment ions at *m/z* 283 [M-H-H_2_O]^−^ and 229 [M-H-CO_2_-CO]^−^, which were formed from precursor ion at *m/z* 301, suggested the presence of ellagic acid. Compared with standard compounds and the literature (29), peak 14 was unambiguously assigned as ellagic acid.

**Table 1:** Chromatographic and mass characteristics of MMP-2 binding ligands.

**No**.	**t_**R**_ (min)**	**MS^**2**^**	**Formula**	**ESI-MS(-)**		**Identification**
				**Measured mass [M-H]^−^or [M+HCOO]^−^**	**Error (ppm)**	
4	8.22	**300.9981**	C_20_H_22_O_11_	437.1110	5.9	Oreganol A
6	8.63	**301.0006**	C_27_H_22_O_18_	633.0767	6.2	Corilagin
11	12.25	451.3176, **301.0107**	C_24_H_50_O_10_	497.3368	8.4	—
14	13.53	**283.9986**, 229.0143	C_14_H_6_O_8_	300.9991	2.3	Ellagic acid
15	13.87	301.0062, **271.0983**	C_21_H_28_O_9_	423.1655	0.0	—
16	15.28	**677.4960**, 451.3290	C_37_H_58_O_11_	723.5026	−4.3	Zebirioside O
17	15.75	**503.3400**	C_36_H_58_O_11_	711.4001	−5.8	Niga-ichigoside F_1_
20	18.25	**487.3368**	C_36_H_58_O_10_	695.3994	−1.9	Peduncloside
21	18.68	**487.3402**	C_36_H_58_O_10_	695.3974	3.7	Rosamultin
22	19.25	**487.3430**	C_36_H_58_O_10_	695.3992	2.2	Kajiichigoside F_1_
23	20.32	**485.3228**, 309.1722	C_36_H_56_O_10_	693.3849	−0.1	Potentillanoside A
24	20.67	647.3819, **485.3219**	C_36_H_56_O_10_	693.3844	−0.9	Quadranoside VIII
28	23.30	184.9697, **112.9872**	C_15_H_22_O_2_	233.1545	1.3	Valerenic acid
29	23.49	**483.3058**, 465.2987	C_30_H_46_O_6_	501.3221	1.0	Medicagenic acid

*The bold values indicate the abundance of the ion is the largest*.

Peak 16 showed an [M+HCOO]^−^ ion at *m/z* 723 and produced fragment ion at *m/z* 677 [M–H]^−^ and *m/z* 485 in negative MS^2^ mode. Compared with standard and the literature (30), peak 16 was deduced as zebirioside O. The [M-162-46-H]^−^ ion in the MS^2^ spectrum of peaks 17, 20, 21, 22, 23, and 24 corresponding to the presence of glucose group in the compounds. The formula of the six compounds was determined to be C_36_H_58_O_11_, C_36_H_58_O_10_, and C_36_H_56_O_10_, indicating their structures were similar. Compared with the pieces of literature (31–36) and standard substances, peaks 17, 20, 21, 22, 23, and 24 were tentatively identified as niga-ichigoside F_1_, peduncloside, rosamultin, kajiichigoside F_1_, potentillanoside A, and quadranoside VIII, respectively. The formula of peaks 28 and 29 was calculated as C_15_H_22_O_2_ and C_30_H_46_O_6_. The two peaks were plausibly assigned as valerenic acid and medicagenic acid as compared with standards and the literature (37).

### Matrix Metalloproteinase-2 Inhibitory Assay

A total of twelve binding ligands were evaluated for their inhibitory activities against matrix metalloproteinase-2 by using the conventional inhibitory assay. The IC_50_ value of batimastat was determined to be 4.58 nM, which was consistent with the literature (38). The assay data of binding ligands are shown in Table 2. The MMP-2 inhibitory activities of the twelve ligands were decreased as follows: niga-ichigoside F_1_, rosamultin, kajiichigoside F_1_, zebirioside O, peduncloside, potentillanoside A, quadranoside VIII, corilagin, oreganol A, ellagic acid, medicagenic acid, and valerenic acid. Except for ellagic acid, the matrix metalloproteinase-2 inhibitory effects of the other eleven ligands were reported for the first time.

**Table 2:** Inhibitory effects of MMP-2 binding ligands.

**No**.	**Compound**	**IC_50_ ±SD(μM)**	**Affinity (kcal/mol)**
4	Oreganol A	30.83 ± 2.13	−8.3
6	Corilagin	28.56 ± 2.83	−9.6
14	Ellagic acid	37.80 ± 1.96	−9.0
16	Zebirioside O	23.19 ± 4.49	−8.4
17	Niga-ichigoside F_1_	20.12 ± 1.55	−7.9
20	Peduncloside	23.24 ± 2.53	−8.6
21	Rosamultin	20.68 ± 2.06	−9.7
22	Kajiichigoside F_1_	22.04 ± 2.15	−9.4
23	Potentillanoside A	25.54 ± 3.01	−8.1
24	Quadranoside VIII	25.88 ± 5.66	−8.3
28	Valerenic acid	82.49 ± 8.87	−7.1
29	Medicogenic acid	64.07 ± 13.60	−8.4
Control	Batimastat	4.58 ± 0.44 nM	—

### Molecular Docking

Three-dimensional pictures of the best-docked conformation of MMP2-rosamultin, MMP2-corilagin, and MMP2-kajiichigoside F1 complexes were presented in Supplementary Figure 9. The binding affinity between rosamultin and MMP2 protein is determined to be −9.7 kcal/mol, which proves to have a good binding effect. Rosamultin interacts with the MMP-2 protein by forming hydrogen bonds with MET-373, CYS-390, PHE-512, ALA-510, ASP-188, and LYS-372, the lengths of which are 2.7, 2.8, 3.0, 2.9, 3.1, and 3.1 Å, respectively. Moreover, the binding affinity between corilagin and MMP-2 is −9.6 kcal/mol. Corilagin interacts with the MMP-2 protein through the formation of hydrogen bonds with VAL-107, TYR-110, LYS-62, PRO-183, HIS-193, and GLY-103, the lengths of which are 3.0, 2.9, 2.7, 3.0, 3.6, 2.8, and 2.8 Å. Furthermore, the binding affinity between kajiichigoside-F1 and MMP-2 protein is −9.4 kcal/mol. Kajiichigoside-F1 interacts with the MMP2 protein through the formation of hydrogen bonds with THR-511, ALA-510, SER-546, MET-373, CYS-390, and ASP-188, the lengths of which are 3.0, 2.9, 3.2, 2.7, 2.7, and 3.0 Å, respectively.

### Comparisons With Previous Ligand Fishing Methods

Several ligand fishing methods have been reported, namely, monolithic column coated with white blood cell membranes (39), Hsp 90α functionalized InP/ZnS QDs embedded mesoporous nanoparticles (13), porcine pancreatic lipase immobilized organic framework UiO-66-NH_2_ (40), and so on. A comparison between the previous ligand fishing method and this work was displayed in Table 3. Compared with the traditional ligand fishing method, this method takes less time and effectively reduces the target protein and solvent expenditure. However, the microfluidic-based ligand fishing system also has certain limitations. For instance, the oil phase (n-hexadecane) will freeze at a low temperature, which will lead to the blockage of the chip pipeline, thereby affecting the ligand fishing result. Therefore, the temperature control system should be used to ensure the accuracy of the experimental results. Our results showed that this microfluidic-based ligand fishing method can be effectively applied to screen for bioactive ingredients from natural products.

**Table 3:** A comparison between the previous ligand fishing method and this work.

**Method**	**Monolithic column -based method**	**Quantum dots embedded mesoporous nanoparticles -based method**	**Metal-organic framework base method**	**Microfluidic chip-based method**
Consumption of organic solvent	AIBN, GMA, EDMA, cyclohexanol and dodecanol	TEOS, CTAB, TMB, APS, Glutaraldehyde	ZrCl_4_, ATA, HAc, HCl, DMF	EDC, NHS, PEI
Consumption of time	Several days	Several days	Several days	Two days
Environmentally friendly	No	No	No	Yes
Cost	Expensive	Moderate	Moderate	Cheap
Enzyme function	Function	Function	Function	Function
Reference	(39)	(13)	(40)	This work

## Conclusion

In this article, a rapid microfluidic chip-based ligand fishing platform for discovering matrix metalloproteinase-2 inhibitors from *R. roxburghii* was presented. Six parameters were investigated to effectively optimize the ligand fishing procedure. A total of eleven new matrix metalloproteinase-2 inhibitors were discovered from the extract of *R. roxburghii*. These inhibitors may contribute to the antitumor effect of *R. roxburghii*. The proposed microfluidic chip-based ligand fishing method offers a good alternative to other screening assays and will pave the way for high-throughput screening from natural products.

## Data Availability Statement

The original contributions presented in the study are included in the article/Supplementary Material, further inquiries can be directed to the corresponding authors.

## Author Contributions

YT contributed to writing, reviewing, editing, funding acquisition, and project administration. MP performed methodology, validation, formal analysis, investigation, and data curation. FZ was involved in writing original draft preparation. QL was involved in formal analysis. PW did the conceptualization and funding acquisition. All authors contributed to the article and approved the submitted version.

## Funding

Financial support was gratefully acknowledged from the National Natural Science Foundation for the Youth (No. 81703701) and the Natural Science Foundation of Zhejiang Province (No. Y21H280036).

## Conflict of Interest

The authors declare that the research was conducted in the absence of any commercial or financial relationships that could be construed as a potential conflict of interest.

## Publisher's Note

All claims expressed in this article are solely those of the authors and do not necessarily represent those of their affiliated organizations, or those of the publisher, the editors and the reviewers. Any product that may be evaluated in this article, or claim that may be made by its manufacturer, is not guaranteed or endorsed by the publisher.

## Supplementary Material

Supplementary Figure 1 Homemade microfluidic system and microfluidic chip.

Supplementary Figure 2 FT-IR of magnetic microspheres with different coats.

Supplementary Figure 3 Zeta potential values of magnetic microspheres with different coats.

Supplementary Figure 4 XRD images of magnetic microspheres and MS@PEI@QDs@PEI@ MMP-2.

Supplementary Figure 5 Effect of amount of magnetic microspheres on the percentage of conjugated MMP-2.

Supplementary Figure 6 Confocal image of MS@PEI@QDs@PEI@MMP-2.

Supplementary Figure 7 Specificity of the microfluidic-based screening system. 3.85 min: loganic acid, 6.35 min: ellagic acid.

Supplementary Figure 8 Chemical structures of MMP-2 binding ligands.

Supplementary Figure 9 3D pictures of the best-docked conformation of MMP2-rosamultin, MMP2-corilagin, and MMP2-kajiichigoside F1 complexes.
