# Supplementary figures and images for: Construction of a Microfluidic Platform With Core-Shell CdSSe@ZnS Quantum Dot-Encoded Superparamagnetic Iron Oxide Microspheres for Screening and Locating Matrix Metalloproteinase-2 Inhibitors From Fruits of Rosa roxburghii
Source: Front Nutr. 2022 Apr 14;9:869528. doi: 10.3389/fnut.2022.869528 (PMC9046974; doi:10.3389/fnut.2022.869528)

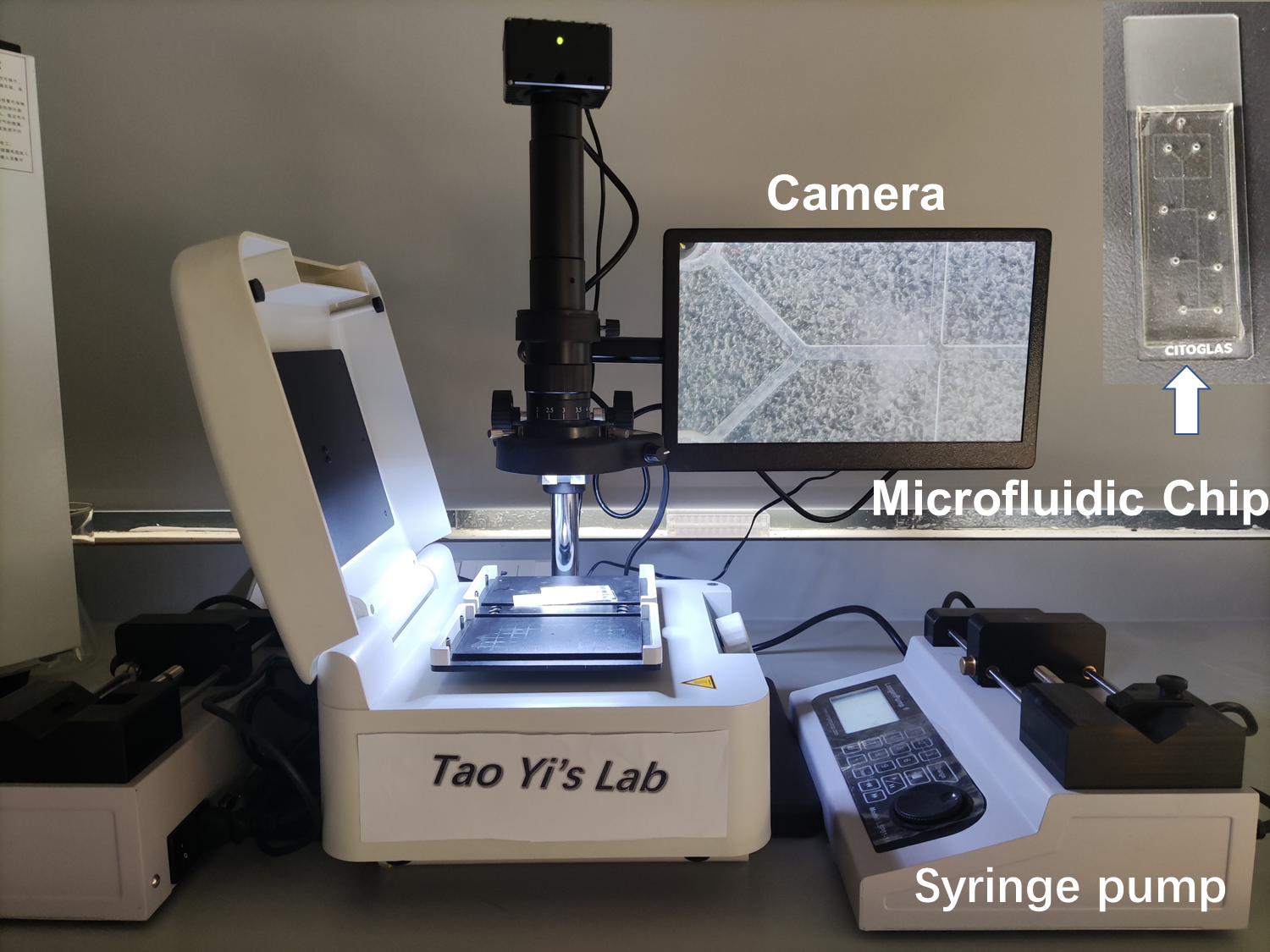

Supplement: Supplementary Figure 1 — Homemade microfluidic system and microfluidic chip. [file Image_1.tiff]

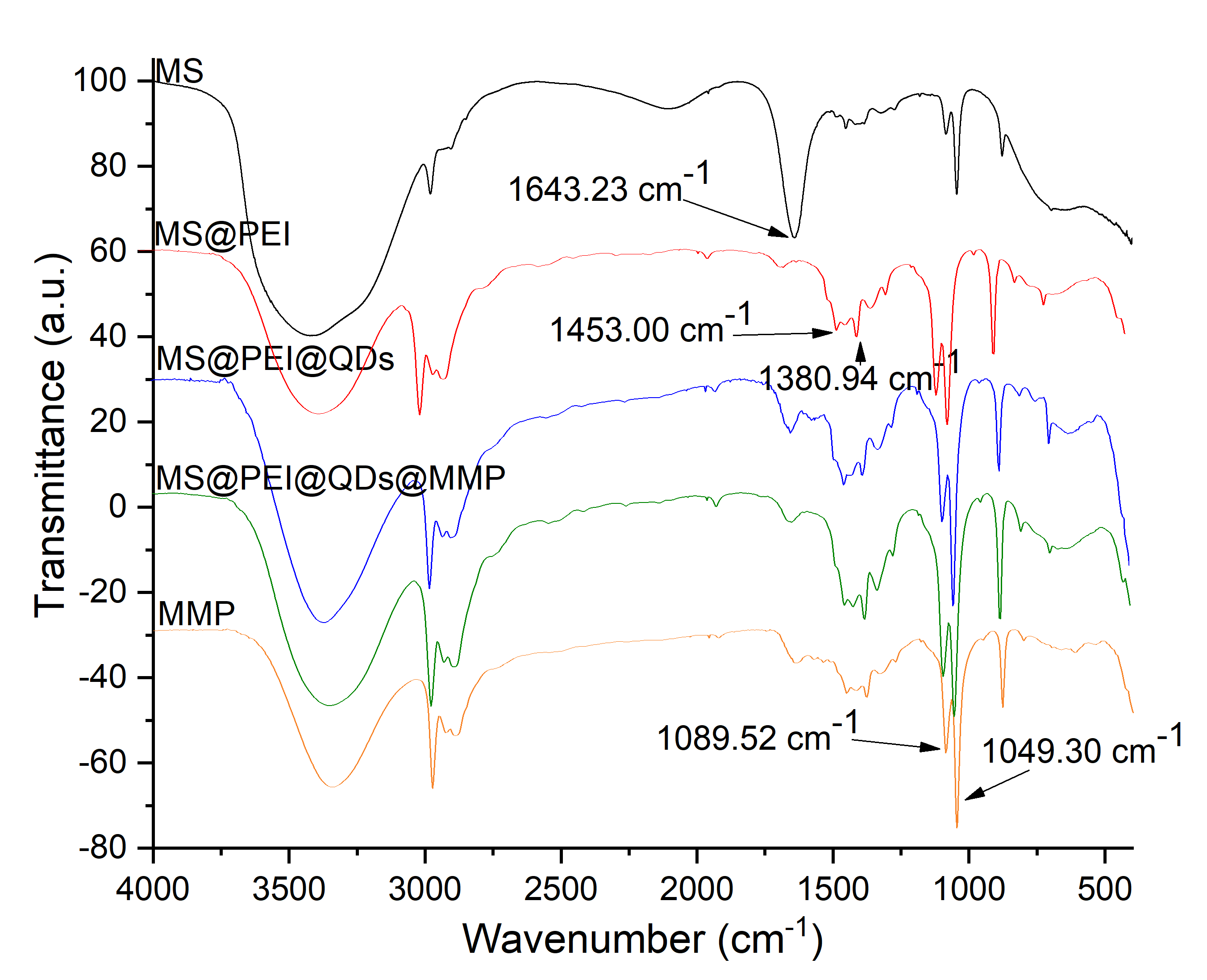

Supplement: Supplementary Figure 2 — FT-IR of magnetic microspheres with different coats. [file Image_2.tif]

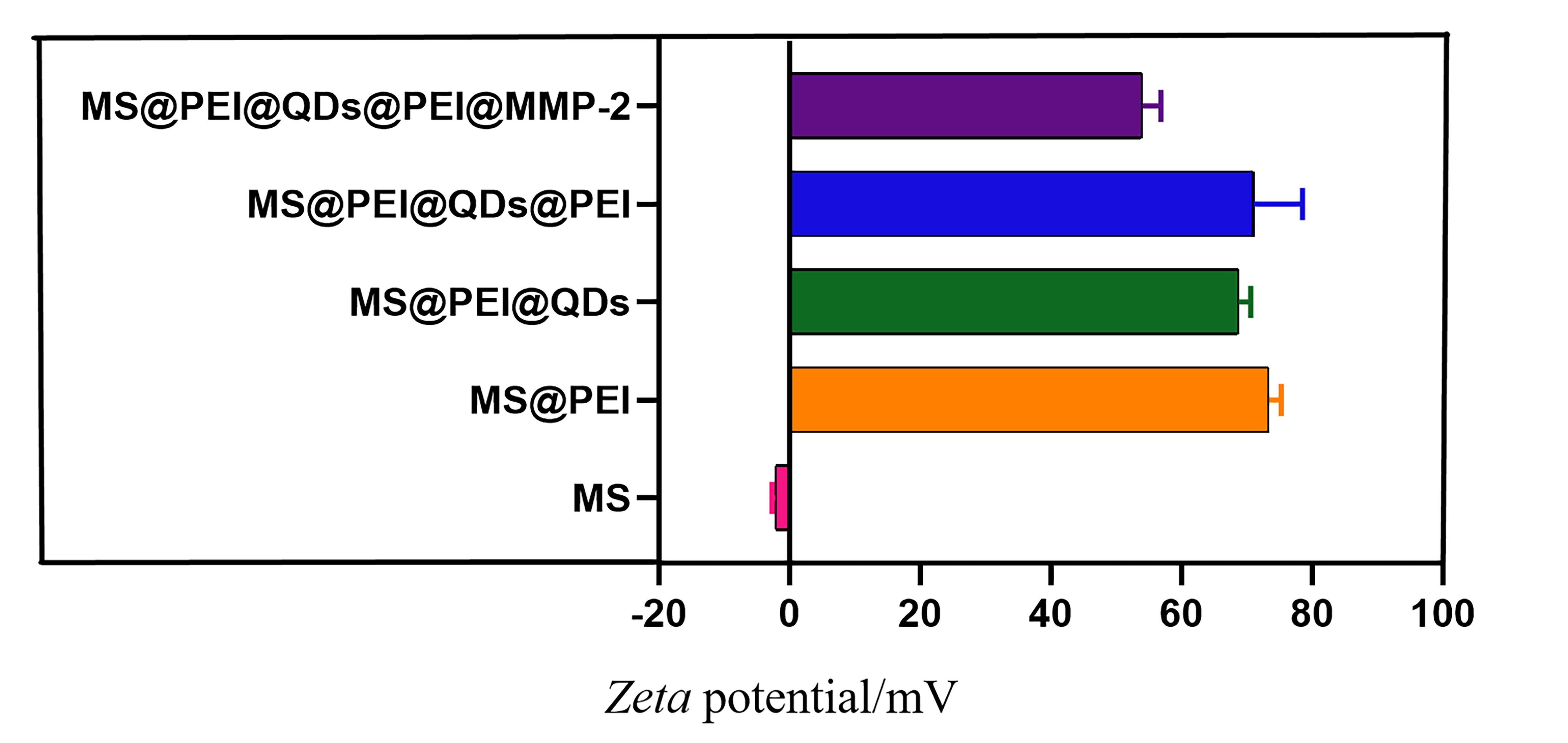

Supplement: Supplementary Figure 3 — Zeta potential values of magnetic microspheres with different coats. [file Image_3.tif]

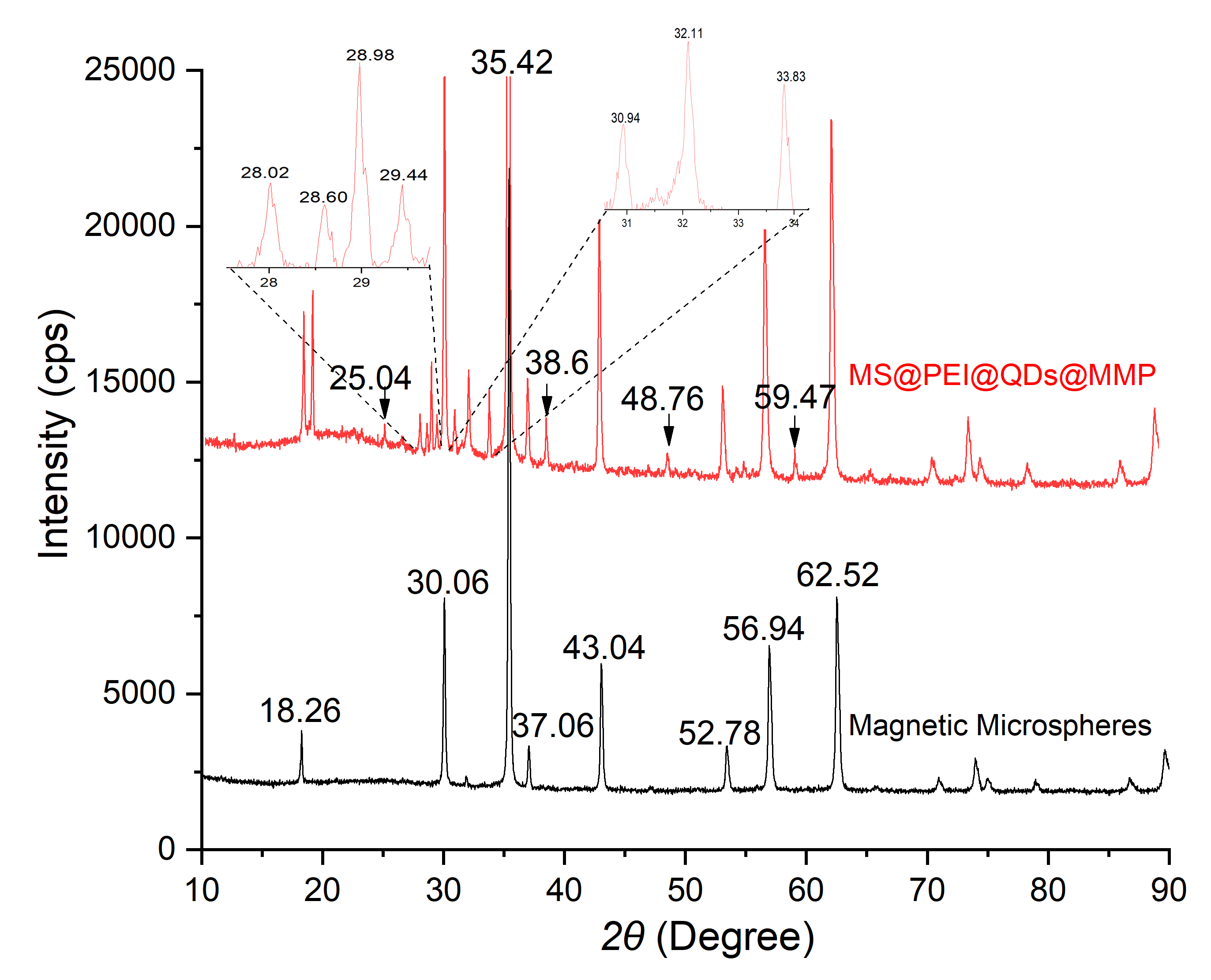

Supplement: Supplementary Figure 4 — XRD images of magnetic microspheres and MS@PEI@QDs@PEI@ MMP-2. [file Image_4.tif]

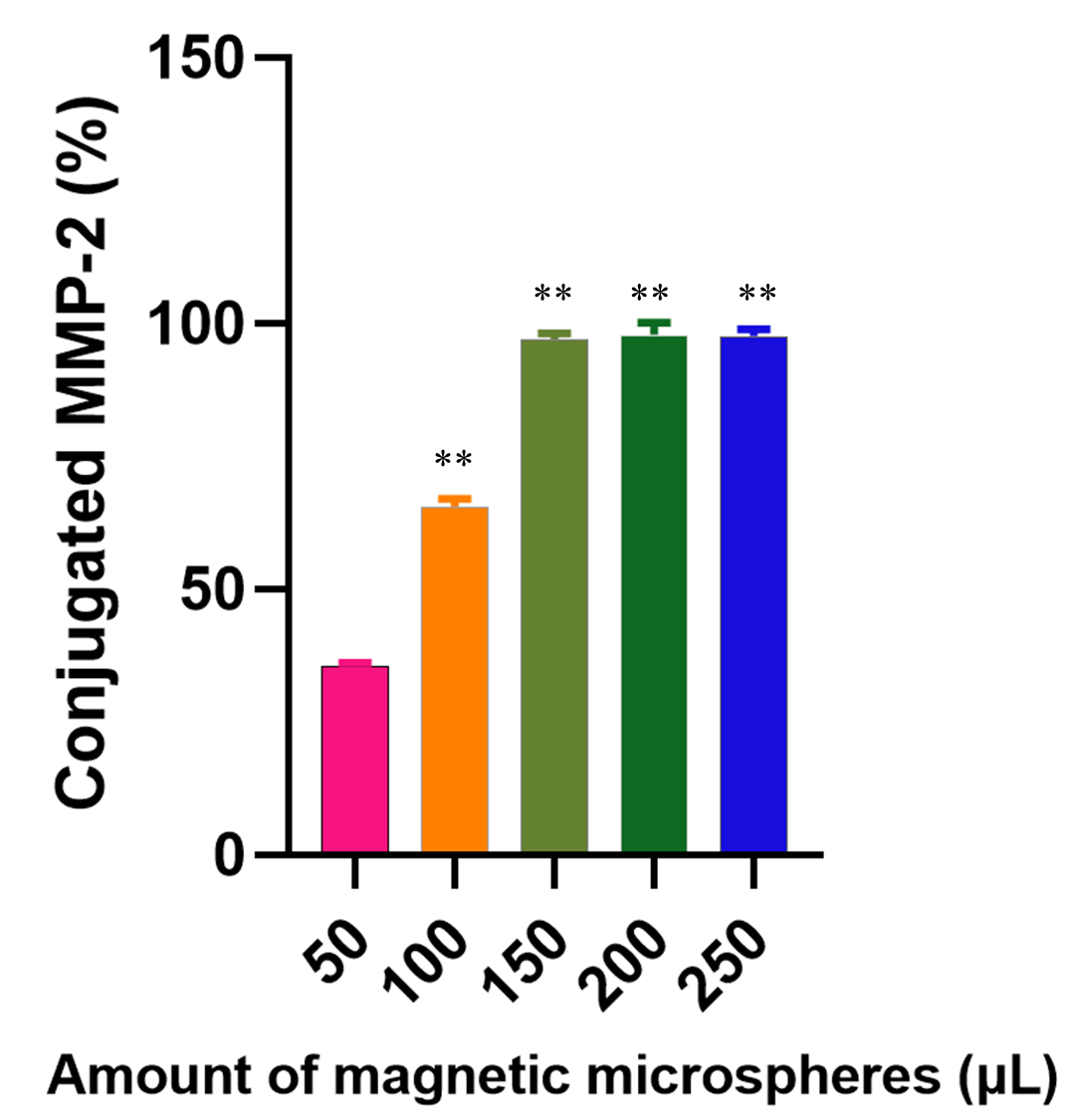

Supplement: Supplementary Figure 5 — Effect of amount of magnetic microspheres on the percentage of conjugated MMP-2. [file Image_5.tiff]

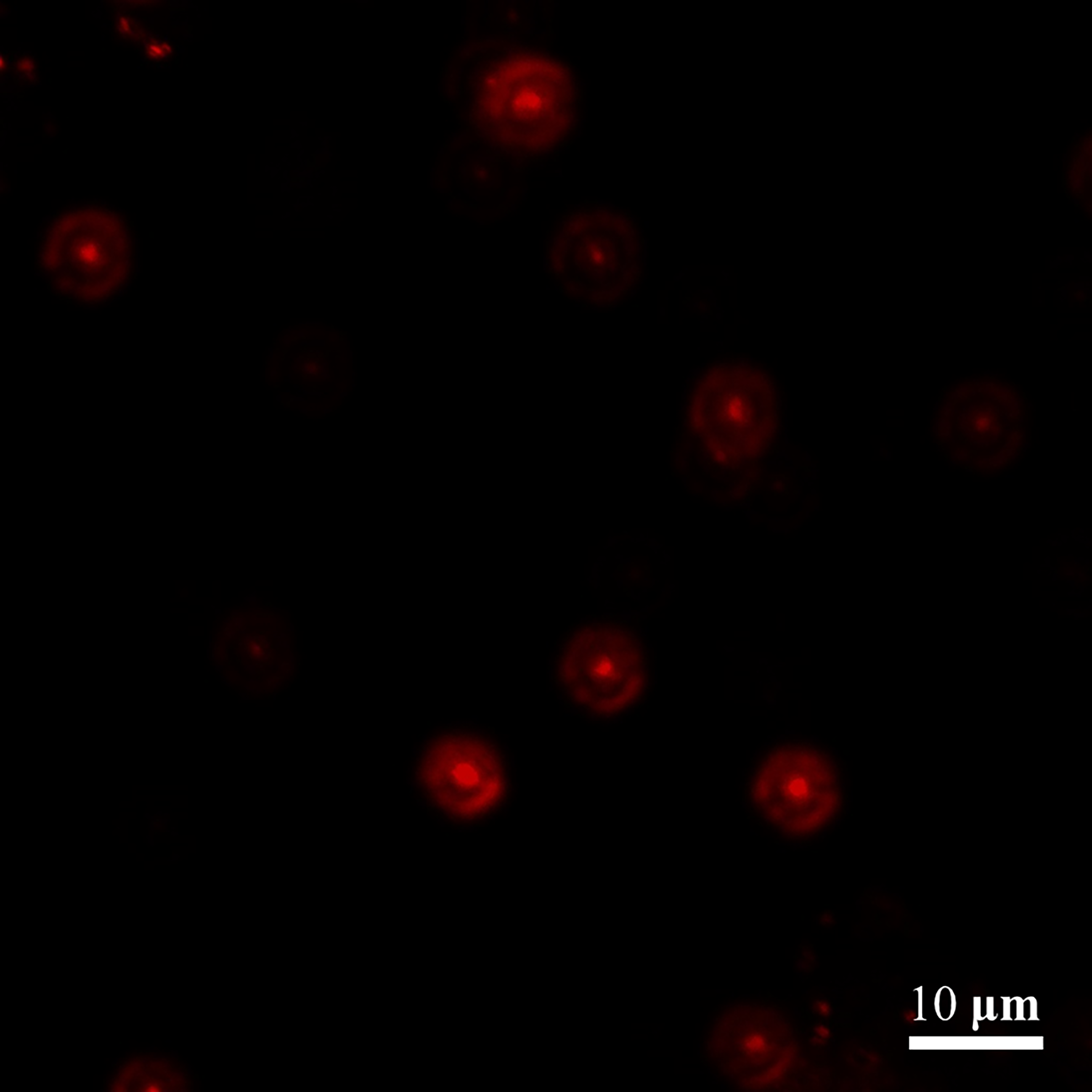

Supplement: Supplementary Figure 6 — Confocal image of MS@PEI@QDs@PEI@MMP-2. [file Image_6.tiff]

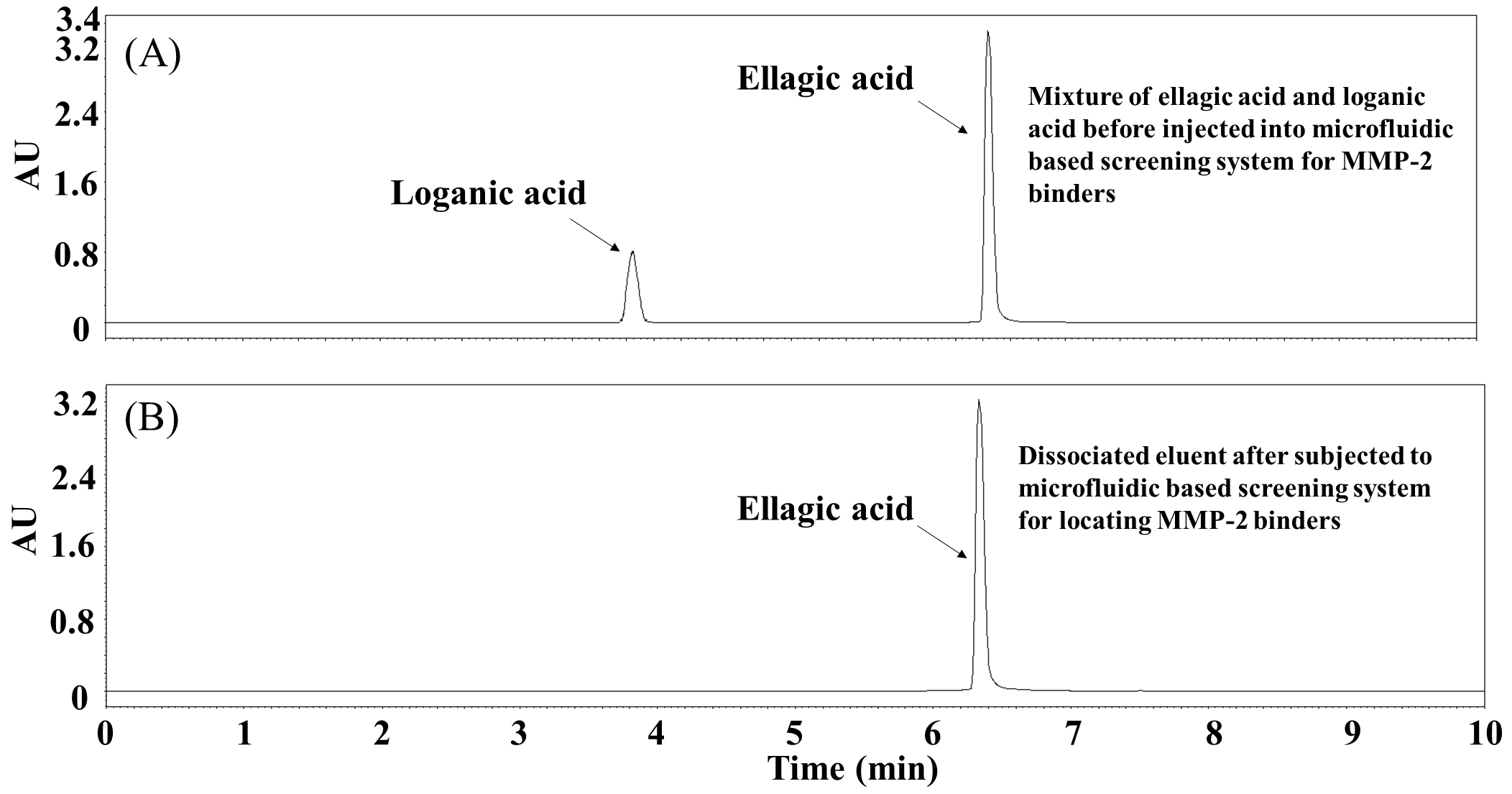

Supplement: Supplementary Figure 7 — Specificity of the microfluidic-based screening system. 3.85 min: loganic acid, 6.35 min: ellagic acid. [file Image_7.tiff]

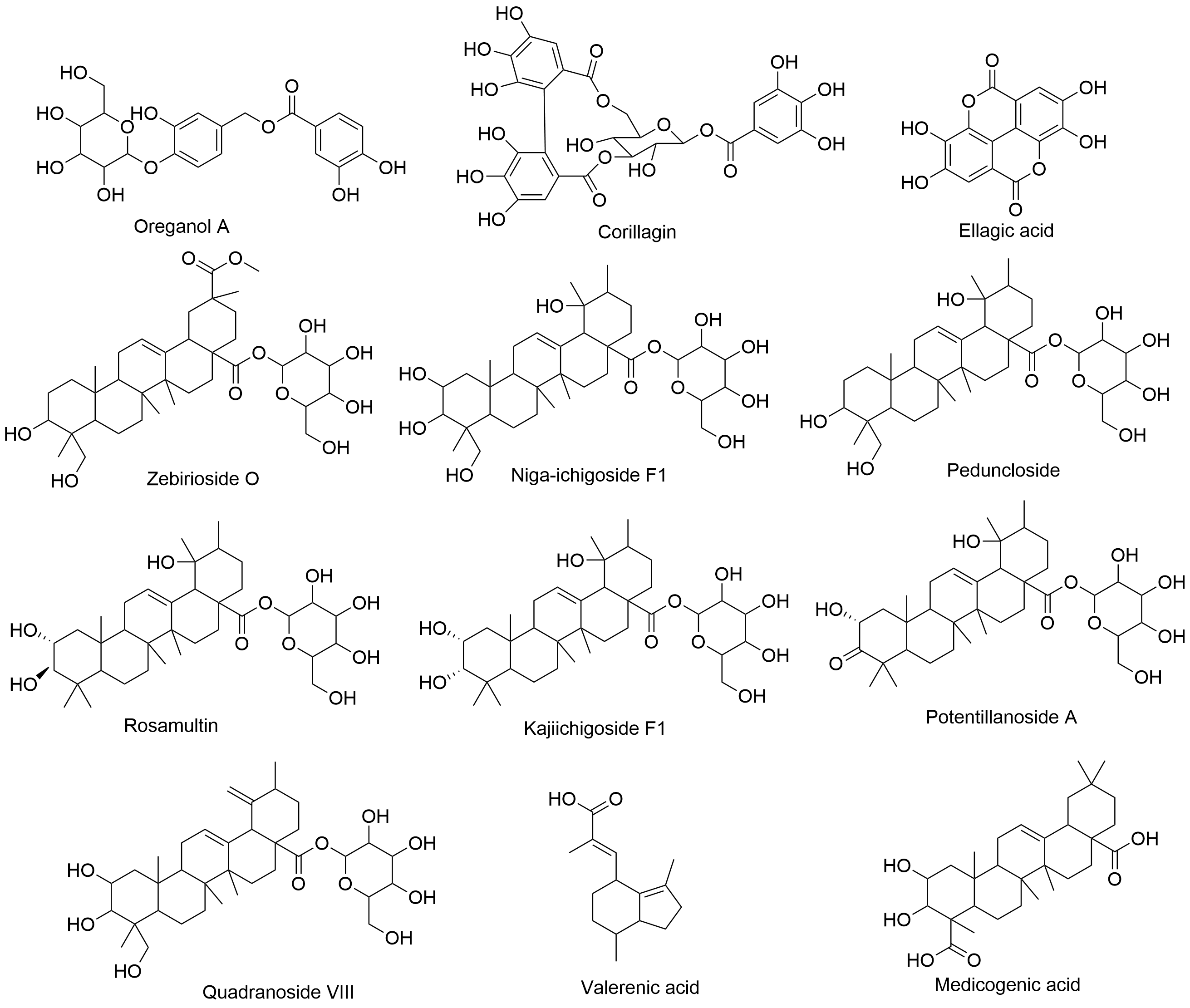

Supplement: Supplementary Figure 8 — Chemical structures of MMP-2 binding ligands. [file Image_8.tif]

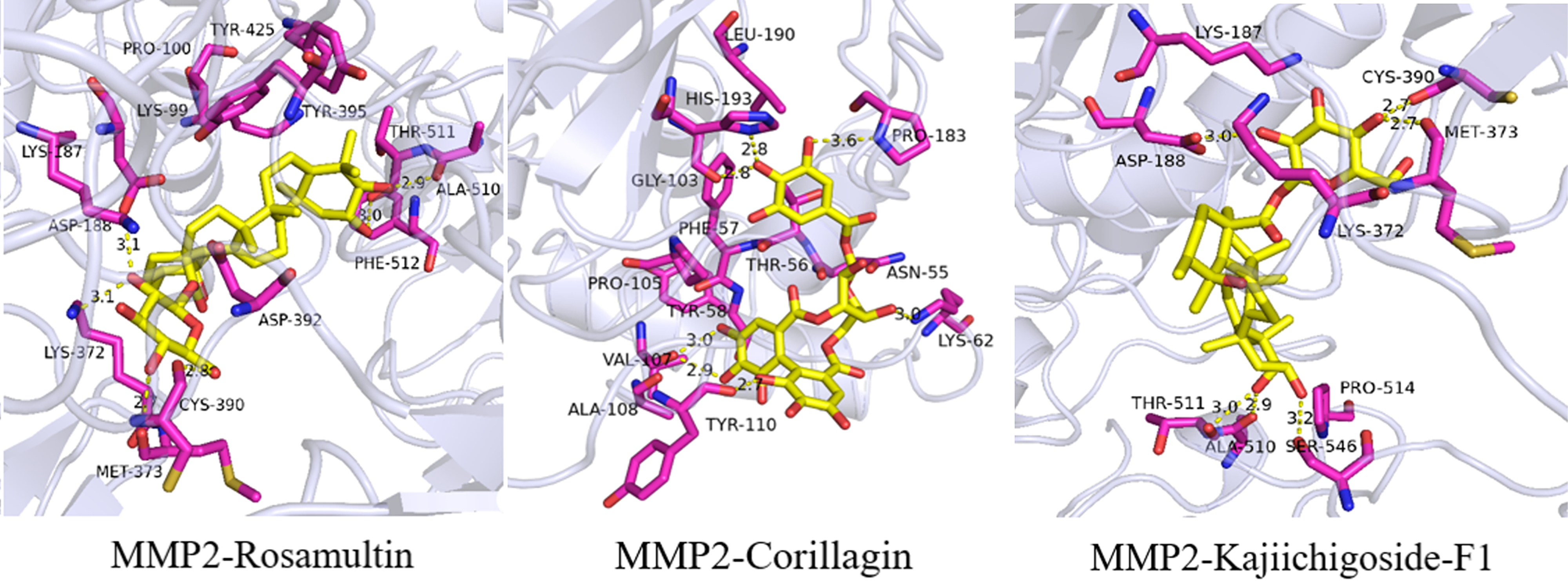

Supplement: Supplementary Figure 9 — 3D pictures of the best-docked conformation of MMP2-rosamultin, MMP2-corilagin, and MMP2-kajiichigoside F1 complexes. [file Image_9.tiff]
